# Detritus Quality Controls Macrophyte Decomposition under Different Nutrient Concentrations in a Eutrophic Shallow Lake, North China

**DOI:** 10.1371/journal.pone.0042042

**Published:** 2012-07-26

**Authors:** Xia Li, Baoshan Cui, Qichun Yang, Hanqin Tian, Yan Lan, Tingting Wang, Zhen Han

**Affiliations:** 1 State Key Joint Laboratory of Environmental Simulation and Pollution Control, School of Environment, Beijing Normal University, Beijing, China; 2 Ecosystem Dynamics and Global Ecology (EDGE) Laboratory, School of Forestry and Wildlife Sciences, Auburn University, Auburn, Alabama, United States of America; 3 International Center for Climate and Global Change Research, Auburn University, Auburn, Alabama, United States of America; Utrecht University, The Netherlands

## Abstract

Macrophyte decomposition is important for carbon and nutrient cycling in lake ecosystems. Currently, little is known about how this process responds to detritus quality and water nutrient conditions in eutrophic shallow lakes in which incomplete decomposition of detritus accelerates the lake terrestrialization process. In this study, we investigated the effects of detritus quality and water nutrient concentrations on macrophyte decomposition in Lake Baiyangdian, China, by analyzing the decomposition of three major aquatic plants at three sites with different pollution intensities (low, medium, and high pollution sites). Detritus quality refers to detritus nutrient contents as well as C∶N, C∶P, and N∶P mass ratios in this study. Effects of detritus mixtures were tested by combining pairs of representative macrophytes at ratios of 75∶25, 50∶50 and 25∶75 (mass basis). The results indicate that the influence of species types on decomposition was stronger than that of site conditions. Correlation analysis showed that mass losses at the end of the experimental period were significantly controlled by initial detritus chemistry, especially by the initial phosphorus (P) content, carbon to nitrogen (C∶N), and carbon to phosphorus (C∶P) mass ratios in the detritus. The decomposition processes were also influenced by water chemistry. The NO_3_-N and NH_4_-N concentrations in the lake water retarded detritus mass loss at the low and high pollution sites, respectively. Net P mineralization in detritus was observed at all sites and detritus P release at the high pollution site was slower than at the other two sites. Nonadditive effects of mixtures tended to be species specific due to the different nutrient contents in each species. Results suggest that the nonadditive effects varied significantly among different sites, indicating that interactions between the detritus quality in species mixtures and site water chemistry may be another driver controlling decomposition in eutrophic shallow lakes.

## Introduction

The decomposition of aquatic macrophytes is an essential process for carbon (C) and nutrient cycling in aquatic ecosystems [1]. Breakdown of aquatic plant detritus significantly mobilizes organic compounds and liberates C in the forms of CO_2_ and dissolved organic carbon. Nutrients released from the detritus decomposition increase nutrient availability in aquatic ecosystems [2], [3]. However, incomplete decomposition of aquatic detritus usually leads to sediment accumulation and increases carbon storage [4], [5], which potentially influences the structure and functioning of lake ecosystems. These effects are especially serious for eutrophic shallow lakes, where high nutrient availability in water bodies and elevated sediment accumulation in lake bottoms may favor overgrowth of macrophytes and accelerate lake terrestrialization [6], [7], [8]. Therefore, studies addressing the main factors that influence macrophyte decomposition in lakes are critical for gaining a fundamental understanding of nutrient cycling and lake succession of eutrophic shallow lakes.

Among the factors that control decomposition processes, detritus nutrient quality plays a major role [5], [9], [10]. Interspecific variations in macrophytes usually result in differences in detritus decomposition rates. In general, detritus with high initial nitrogen (N) and phosphorus (P) contents as well as low C∶N, C∶P, and N∶P ratios is considered to have high detritus quality. This kind of detritus decomposes faster in ecosystems than low quality detritus [11]–[14]. In natural ecosystems, most detritus is composed of multiple species, and detritus decomposes as a mixture of species rather than as single species. As a result, the decomposition rates of species mixtures may deviate significantly from the expected rates based on additive mass losses of single species because of the chemical interactions between detritus of different species [15]–[17].

Physical and chemical conditions surrounding plant detritus during decomposition are additional factors that control macrophyte decomposition [9], [10], [18]. In an aquatic ecosystem where moisture is not constraining, detritus decomposition is significantly influenced by temperature, nutrient availability and pH levels [19], [20]. It has been widely recognized that aquatic ecosystems with high concentrations of dissolved nutrients tend to exhibit rapid detritus decomposition [21], [22]. In some cases, high nutrient concentrations play greater roles than do the quality of detritus in influencing decomposition rates [1], [23]. However, due to effects of high detritus quality and variations in microbial compositions and activities, some other studies suggest that high nutrient levels in water may not stimulate detritus decomposition [19], [24]–[26].

Despite widespread concerns about detritus decomposition in aquatic ecosystems, few studies have explored the factors regulating this process in eutrophic lakes. Detritus of macrophyte from nutrient-enriched sites is often characterized by a high nutrient content as a result of the elevated water nutrient concentrations [14], [27]. Nutrients provided by detritus are the major nutrient sources for decomposers in these ecosystems [28]. The interactions that lead to nonadditive effects in species mixtures also aid detritus decomposition through the transfer of nutrients from liable to recalcitrant detritus [17], [29], [30]. The role of nutrient conditions of the ambient environment on detritus decomposition is still unclear. Nutrients in water may either accelerate decomposition or impede decomposition by synthesizing secondary compounds in detritus, such as lignin and phenolic, forming hard-to-degrade complexes [16], [26]. The spatial heterogeneity of water nutrient levels in eutrophic lakes makes them ideal test sites to explore how detritus decomposition responds to different nutrient conditions. Therefore, investigating the effects of detritus quality on decomposition under different nutrient concentrations in eutrophic lake is necessary and will enrich our understanding of how different factors regulate detritus decomposition and accumulation.

In the past 30 years, increasing nutrient inputs and water consumption have created favorable conditions for aquatic macrophytes in Lake Baiyangdian. As a result, aquatic plants have spread quickly and cover a large proportion of the lake. Additionally, incomplete decomposition of dead plants has further decreased water levels and resulted in positive feedbacks for the growth of macrophytes [7], [8]. To reveal the role of macrophyte decomposition in C accumulation and nutrient cycling in the lake, we investigated the effects of detritus quality on detritus decomposition in samples of single species and species mixtures under three nutrient concentrations in a period of 270 days. We also observed the dynamics of N and P released from detritus during the study period. We hypothesized that in Lake Baiyangdian: (1) increases in nutrient concentrations in the lake water due to human disturbance allow rapid decomposition to occur; (2) macrophyte species has a stronger effect on decomposition than site conditions; and (3) decomposition of species mixtures is influenced by species types, mass ratios of component species and water nutrient conditions. Long-term decomposition is not addressed in this study because the nutrient releases and mass losses caused by decomposition become very slow after one year or, in some cases, even less than one year [31]. The aims of this study are as follows: (1) to study roles of water nutrient concentrations and detritus quality in the macrophyte decomposition in Lake Baiyangdian; (2) to test nonadditive effects in decomposition of species mixtures by utilizing different ratios of species in mixtures under various nutrient conditions; and (3) to illustrate the dynamics of elements (C, N, and P) for different sites and detritus compositions during decomposition.

## Methods

### Ethics statement

No specific permits were required for the study area or activities. Lake Baiyangdian is owned by the Chinese government. Our study sites do not contain any strictly protected areas, or endangered or protected species.

### Study area

Lake Baiyangdian is the largest freshwater inland lake in North China and covers latitudes from 37°43′N to 39°02′N and longitudes from 115°45′E to 116°07′E. The climate in this area is characterized as a temperate continental monsoon climate with mean annual precipitation of 510 mm. The total area of the lake is 366 km^2^ when its average water depth is 2.5 m. Since the 1980s, part of Lake Baiyangdian has been drying up, as the water level has declined sharply [32]. In addition, nutrient pollution from adjacent farmlands and residential areas has greatly enriched the nutrient levels in the lake, resulting in serious eutrophication. Elevated nutrient levels favor overgrowth of aquatic macrophytes, leading to increases in sediment accumulation, which further stimulates plant growth and accelerates the succession of the lake ecosystem.

### Design of decomposition experiments

We performed decomposition experiments at three sites in Lake Baiyangdian: Caiputai, Shaochedian, and Dazhangzhuang. To demonstrate the effects of water nutrient conditions on decomposition, the sites were selected based on their exhibition of different pollution intensities. Caiputai (low pollution site) is a site where there is little disturbance or nutrient pollution. Shaochedian (medium pollution site) is influenced by non-point source pollution from farm land and point source nutrient pollution from adjacent villages. Dazhangzhuang (high pollution site) is the site subjected to the most severe human disturbances and nutrient pollution among the three sites because of the intensive farming activities and dense population around the site. The dominant species of emergent macrophytes, floating macrophytes, and submersed macrophytes in the lake are *Phragmites australis* (*P. a.*), *Nelumbo nucifera* (*N. n.*), and *Potamogeton pectinatus L.* (*P. p.*), respectively. Thus, we chose these three species as the representative macrophytes to test their decomposition. Detritus of these plant species was collected from the three sites on 24 September, 2009, when the plants began to senesce. The detritus samples were air dried at room temperature for 2 weeks in order to reach a constant weight. To keep consistency of samples placed at different sites, we mixed together 1500 g of air-dried detritus for each species collected at the three sites (500 g for each site) to reduce differences in detritus quality caused by spatial variations among the sites.

Two kinds of decomposition experiments were conducted in this study: single species experiments, in which the decomposition rate of each species was measured; and species mixture experiments, where we studied decomposition processes in detritus mixtures. For the latter type of experiment, combinations of two of the three species (*P. a.* and *N. n.*, *P. a.* and *P. p.*, or *N. n.* and *P. p.*) were mixed together at ratios of 75∶25, 50∶50, and 25∶75 (mass basis) to study their decomposition processes. For both types of experiments, 5 g of air-dried detritus consisting either of a single species or a species mixture was loaded into 0.5 mm mesh litterbags (15 cm×15 cm). In total, 864 bags were prepared for 12 kinds of samples (3 single species and 9 species mixtures), and 5 more bags for each species were prepared as initial detritus samples. On 2 December, 2009, we placed 288 bags at each site to allow for three replicates and eight sampling times for the 12 kinds of samples (3×8×12 bags). The remaining 15 bags with detritus of single species were brought back to our laboratory to estimate initial detritus mass and chemical characteristics. All the litterbags used in field experiments were affixed inside square metal cages (5×0.4×0.4 m, mesh wide 5 cm). They were tethered to the frame of the cages and about 15 cm above the lake bottom, which diminished effects of sediment on decomposition. Three replicate litterbags of each kind of detritus were retrieved from each site after 3, 7, 13, 115, 148, 178, 207, and 270 days (the lake was frozen for approximately 3 months after the 13th day of the experiment). The remaining mass of the initial and retrieved detritus samples were measured after samples were washed and subsequently dried in an oven at 60°C for three days. Then the oven-dried detritus samples were ground to a fine powder (100 mesh) and analyzed for the total C, N, and P contents. The total C and N contents in the detritus were analyzed using an Elemental Analyzer (Elementar, Inc., Germany). After digesting in sulfuric acid/hydrogen peroxide, the total P content in the detritus was measured colorimetrically on an AutoAnalyzer (Bran+Luebbe GmbH, Inc., Germany), using the ammonium molybdate ascorbic acid method. Initial nutrient characteristics of mixture species were calculated based on the initial nutrient contents and mass ratios of single species.

### Water chemistry characteristic analyses

The water chemical characteristics at low, medium, and high pollution sites were measured at each sampling time during the experimental period. Water samples were collected 0.2–0.5 m above the sediment with a stainless-steel water sampler. Water pH was measured with a portable Hach pH meter (Hach, Inc., U.S.A.) in the field. Additionally, three water samples from each site were brought to our laboratory in a cooler for water nutrients determination. These samples were filtered through a 0.22 µm filter before measuring nutrient concentrations. The concentrations of NO_3_-N and NH_4_-N were analyzed using an iron chromatograph (Dionex, Inc., U.S.A.). The total phosphorus (TP) concentrations in the water samples were measured with an inductively coupled plasma mass spectrometer (ICAP-9000, Jarre-ASH, Inc., U.S.A.). In this study, water temperature effects were not considered because similar temperatures were observed at the three sites according to a two-sample equal variance *t*-test (*P*>0.19).

### Data analyses

To determine the nonadditive effects in the species mixtures, we defined the expected dry mass remaining at time *t* (days). This parameter was calculated based on the mass ratio of each species and the remaining mass in corresponding single species samples of the component species that were collected at the same site at the same sampling time [33]:


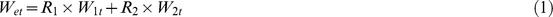


where *R_1_* and *R_2_* are the initial dry mass ratios for species 1 and species 2 in the mixture, respectively, and *W_1t_* and *W_2t_* are the remaining masses of the two single species samples at time *t*.

To evaluate the strength of the nonadditive effects in the species mixtures, the interaction rate (*r_it_*) at time *t* was used and estimated as follows [16], [33]:


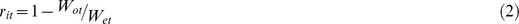


in which *W_ot_* is the observed mass remaining at time *t*. In the equation positive *r_it_* values indicate positive interactions in detritus mixtures and negative *r_it_* value represent negative interactions in mixtures. Additionally, a higher absolute value of *r_it_* indicates a larger difference between observed and expected mass remaining.

The effects of site and species on detritus mass remaining at the end of the experiments were tested using a two-way analysis of variance (ANOVA) with site and species as main effects for single species (3 species). We also performed ANOVA for the species mixtures with site and composition (3 kinds of composition) as main effects. Species evenness (75∶25, 50∶50, 25∶75) was set to nest within composition to test the effect of species evenness on decomposition. Pearson correlation analysis was used to further analyze the effects of the water nutrient conditions on decomposition at the three sites. The mean mass loss rates of all detritus samples were regressed against the mean values of pH, TP, NH_4_-N, and NO_3_-N in the water column at each sampling time for each site. We used a similar Pearson correlation analysis to test the interspecific differences in decomposition by analyzing the averaged values of mass loss at the three sites at each sampling time and the initial mean nutrient levels in the detritus. Paired *t*-tests were used to compare the observed and the expected mass remaining data. The difference of detritus C, N, or P dynamics between single species and mixture species as well as between different sites during the whole experimental period were tested with mixed linear model analyses. All the data (including data for ANOVAs, Pearson correlation, mixed linear model analyses, and Paired *t*-tests) was checked for normality by the Kolmogorov-Smirnov test (n>50) or the Shapiro-Wilk test (n<50) and logistically transformed when needed. The Spearman's correlation analysis was performed when variables were still non-normal after transformation. All of these analyses were carried out using SPSS statistical software (version 17.0).

## Results

### Nutrient conditions at sites and initial detritus quality characteristics

The three study sites had similar climate and dominant aquatic macrophytes, but they varied substantially in water chemistry due to different pollution intensities (Table 1). The mean concentrations of TP, NH_4_-N, and NO_3_-N and averaged pH values in the lake water were highest at the high pollution site, which received a large amount of nutrient pollutions from the adjacent cropland and residential areas. The lowest water nutrients and pH were observed at the low pollution site, where water chemistry was little affected by human activities and the lowest variations of water nutrients were found. A high input of nutrient pollutions from farming activities also occurred at the medium pollution site and high nutrient concentrations were detected during the farming period (late March and April). On the other hand, intra-annual changes in precipitation and temperature are also responsible for the variation of water nutrients levels. For example, low values for the nutrients and pH of the three sites were found from late May to August which is the raining season in our study area.

**Table 1:** Chemical characteristics (mean±SD with ranges in parentheses) of bottom water at study sites in Lake Baiyangdian during the experimental period.

	pH		TP (mg L^−1^)		NH_4_-N (mg L^−1^)		NO_3_-N (mg L^−1^)	
PI	Initial	Mean (Range)	Initial	Mean (Range)	Initial	Mean (Range)	Initial	Mean (Range)
Low	7.3	7.6±0.415	0.004	0.011±0.007	0.000	0.089±0.236	3.128	1.490±1.750
		(7.2–8.3)		(0.004–0.023)		(0.000–0.624)		(0.175–4.466)
Medium	7.8	7.8±0.176	0.072	0.088±0.051	0.000	0.158±0.332	0.611	2.372±3.184
		(7.8–8.1)		(0.072–0.200)		(0.000–0.889)		(0.000–9.440)
High	8.3	8.2±0.261	0.171	0.203±0.077	8.046	3.804±4.648	3.985	5.775±6.333
		(8.1–8.3)		(0.093–0.310)		(0.000–9.827)		(0.000–16.721)

PI is pollution intensity, TP is total phosphorus.

The initial C, N, and P contents differed considerably among the three species (Table 2). *P. a.* exhibited the highest C and initial C∶N, C∶P and N∶P ratios (mass basis) but had lower N and P contents than the other two species. The initial P content in *N. n.* was almost five times greater than that in *P. a.* The initial C∶N, C∶P, and N∶P ratios varied slightly between *N. n.* and *P. p.*, although initial C, N, and P levels were different for the two species (Table 2).

**Table 2:** Initial detritus nutrient characteristics of macrophytes and detritus mass remaining at the end of the experiment at low pollution (Low), medium pollution (Medium) and high pollution (High) sites.

		C (%)		N (%)		P (%)					W (%)		
Species	Ratio	Mean	SD	Mean	SD	Mean	SD	C∶N	C∶P	N∶P	Low	Medium	High
**Single**													
***P. a.***	-	42.502	0.139	0.930	0.227	0.034	0.006	45.675	1239.278	27.132	61.010	57.932	56.669
***N. n.***	-	39.877	0.435	1.808	0.100	0.170	0.011	22.054	235.002	10.656	31.993	36.965	29.507
***P. p.***	-	30.334	0.552	1.357	0.010	0.125	0.031	22.352	242.077	10.830	36.948	37.240	29.792
**Mixture**													
***P. a.*** ** and ** ***N. n.***	75∶25	41.846	-	1.150	-	0.068	-	36.390	614.082	16.875	55.967	53.885	57.817
	50∶50	41.190	-	1.369	-	0.102	-	30.080	403.852	13.426	45.427	47.913	44.297
	25∶75	40.533	-	1.589	-	0.136	-	25.513	298.391	11.696	31.379	36.682	34.030
***P. a.*** ** and ** ***P. p.***	75∶25	39.460	-	1.037	-	0.057	-	38.046	691.693	18.180	54.443	56.886	44.830
	50∶50	36.418	-	1.144	-	0.080	-	31.838	456.359	14.333	52.546	50.926	35.185
	25∶75	33.376	-	1.250	-	0.103	-	26.691	325.448	12.193	52.079	50.134	34.796
***N. n.*** ** and ** ***P. p.***	75∶25	37.491	-	1.695	-	0.159	-	22.114	236.400	10.690	32.359	40.886	26.128
	50∶50	35.106	-	1.583	-	0.147	-	22.182	238.008	10.729	38.705	45.672	28.089
	25∶75	32.720	-	1.470	-	0.136	-	22.260	239.877	10.776	34.124	43.970	25.062
**Mean**		37.571	-	1.365	-	0.110	-	28.766	435.039	13.960	43.914	46.591	37.183
**SD**		4.468	-	0.317	-	0.049	-	7.818	297.024	4.866	10.854	7.624	11.280

W is the percentages of remaining biomass at the end of the experiment; C, N, and P are percentage of C, N, and P based on dry mass; C∶N, C∶P, and N∶P are mass ratios.

Species abbreviations are: *P. a.*, *Phragmites australis*; *N. n.*, *Nelumbo nucifera*; *P. p.*, *Potamogeton pectinatus L.*

### Effects of water chemistry and detritus quality on decomposition

According to ANOVA analysis, detritus decompositions of single species and species mixtures were strongly influenced by both site and species (*P*<0.05, Table 3). However, compared with species type (*P*<0.001), site condition had a relatively weak effect on detritus decomposition (*P* = 0.038) for single species. This result indicates that detritus quality plays the principal role in single species decomposition in this study. For species mixtures, site condition and species composition as well as the evenness of species all had significant effects on detritus decomposition (Site: *P*<0.001; Composition: *P*<0.001; Evenness: *P*<0.001), and a higher *F*-value for the species composition effect (*F* = 18.852) than the site effect (*F* = 12.174) also indicates a stronger detritus quality on decomposition than site condition.

**Table 3:** Effects of site and species on detritus mass remaining for single species and effects of site and species composition and evenness on detritus mass remaining for species mixtures during the experimental period.

Source of variation	Method	MS	F-value	P-value
**Single species**				
Site	Two-way ANOVA	0.015	3.926	0.038
Species		0.174	47.035	<0.001
Site×Species		0.004	1.096	0.389
**Species mixture**				
Site	Two-way ANOVA	1.362	12.174	<0.001
Composition		2.109	18.852	<0.001
Site×Composition		0.403	3.606	0.012
Evenness within composition	Nested ANOVA	0.686	5.878	<0.001

The nutrients and pH in bottom water had significant effects on detritus decomposition, but the regulatory factors differed among sites (Table 4). At the low pollution site, NO_3_-N presented a significant, negative effect on detritus decomposition, while pH had a positive effect on detritus mass loss. However, pH showed a negative correlation with mass loss at the high pollution site. Besides that, NH_4_-N was found to be another significant water chemistry factor, which negatively controlled detritus decomposition at this site. No significant correlations between mass loss and site chemical conditions were observed at the medium pollution site in this study.

**Table 4:** Spearman's correlation coefficients (*r*) between mean detritus mass loss rates and mean site chemical characteristics during the decomposition period.

Site	pH	TP	NH_4_-N	NO_3_-N
Low pollution site	0.855*	0.512	−0.204	−0.750*
Medium pollution site	0.571	0.250	−0.178	−0.630
High pollution site	−0.786*	0.429	−0.815*	−0.523

* *P*<0.05.

TP is total phosphorus.

Regression analyses between detritus decomposition and initial detritus nutrient contents indicate that mass losses of detritus were positively related to the initial detritus N and P contents and negatively related to the C∶N, C∶P, and N∶P mass ratios (Table 5). At an early stage of decomposition, mass loss (<13 days) was slightly controlled by the initial C∶N, C∶P, and P∶N ratios. The effects increased with time, and were strongly correlated with mass loss at the last two sampling times (*r*>0.9, *P*<0.01). Positive correlations were found between detritus P and N contents and mass loss over the entire experimental period (Table 5).

**Table 5:** Pearson correlation coefficients (*r*) between mean detritus mass losses and initial detritus quality factors during the decomposition period.

	Percentage of mass losses at different sampling times							
Factor	3 days	7 days	13 days	115 days	148 days	178 days	207 days	270 days
N content	0.756**	0.705*	0.707*	0.886**	0.881**	0.890**	0.759**	0.829**
P content	0.773**	0.728**	0.705*	0.920**	0.886**	0.943**	0.901**	0.934**
C∶N ratio	−0.600*	−0.561	−0.528	−0.807**	−0.729**	−0.842**	−0.949**	−0.925**
C∶P ratio	−0.605*	−0.567	−0.525	−0.812**	−0.736*	−0.846**	−0.956**	−0.929**
N∶P ratio	−0.559	−0.524	−0.467	−0.783**	−0.710**	−0.817**	−0.945**	−0.906**

* *P*<0.05,

** *P*<0.01.

C∶N, C∶P, N∶P are based on mass ratios.

### Decomposition processes of single species and species mixtures

The decomposition processes for the three macrophyte species present clear differences among the three study sites (Table 2). Detritus mass losses were highest at the high pollution site for all species, while samples collected from the low and medium pollution sites remained similar in detritus mass at the end of the study (two-sample equal variance *t*-test, *P* = 0.0613). At the last sampling time, both the *N. n.* and *P. p.* samples had lost more than 60% of their initial dry mass over the three sites. The *P. a.* samples had lost less than 45% of their initial mass at the end of this study at the three sites.

**Figure 1:**
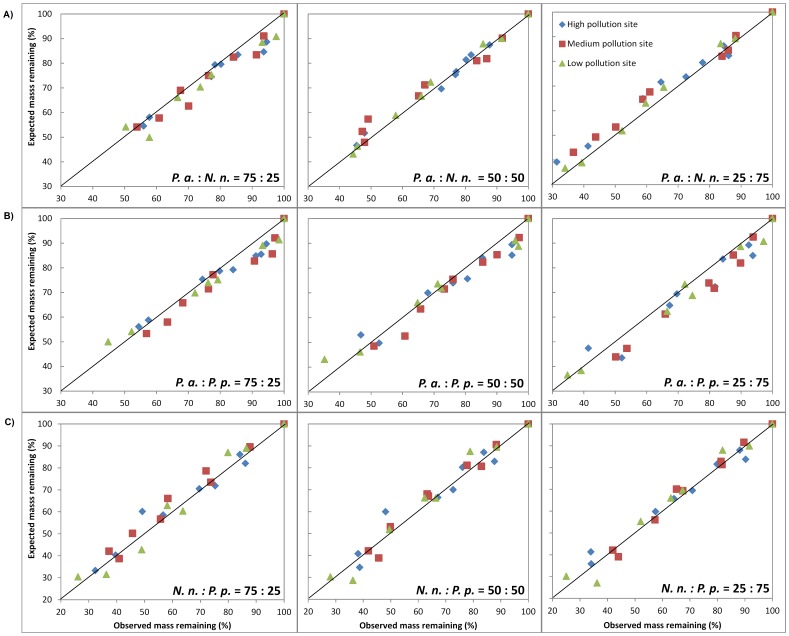
Dry mass remaining in the species mixtures. Mean percentages of observed dry mass remaining (O.) and expected dry mass remaining (E.) in the species mixtures of A) *P. a*. and *N, n.* B) *P. a.* and *P. p.* and C) *N. n.* and *P. p.* over the experimental period at Low, Medium and High pollution sites in Lake Baiyangdian. Deviation from 1∶1 line (solid line) suggests interactions in species mixtures on decomposition (Species abbreviations are, *P. a.*: *Phragmites australis*; *N. n.*: *Nelumbo nucifera*; *P. p.*: *Potamogeton pectinatus L.*).

**Figure 2:**
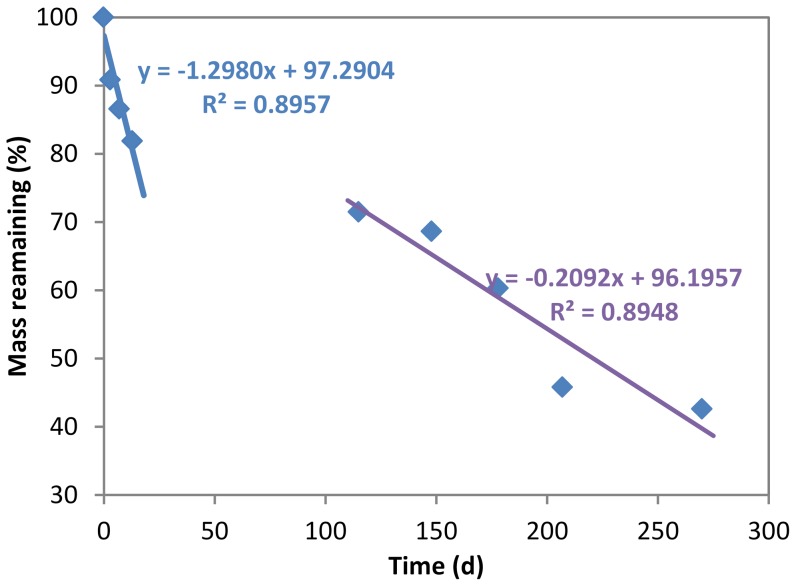
Detritus mass remaining of all the samples. Mean percentage of detritus mass remaining of all the samples during experimental period. The blue and purple solid lines are the linear regression lines for mass remaining at the early decomposition stage and the later decomposition stage, respectively.

Deviations of observed mass remaining from expected mass remaining were observed due to the nonadditive effects of species mixtures on decomposition (Fig. 1). Almost all the observed mass remaining were lower than expected values for species mixtures of *P. a.* and *N. n.* at ratios of 50∶50 and 25∶75, which suggests that positive interactions may exist between these two species (Fig. 1A). For *P. a.* and *P. p.* mixtures, negative interactions occurred because most observed mass remaining values were higher than expected values at the three ratios (Fig. 1B). As to *N. n*. with *P. p* mixtures, observed mass remaining at different mixed ratios is either higher or lower than the corresponding expected values, indicating insignificant interactions between these two species (Fig. 1C). Interaction rates (*r_i_*) in mixtures were further investigated by dividing the whole experimental period into an early stage (before frost) and a later stage (after frost) since detritus mass loss rate after frost (0.2092) was much lower than the previous three sampling times (1.2980) (Fig. 2, Table 6). Species interactions in mixtures were more significant in the later stage than in the early stage. During the first three sampling times, a significant, negative interaction (*P*<0.05) was found in the species mixture of *P. a.* and *P. p.* at a ratio of 75∶25 at the low pollution site. Weak and negative interactions (*P*<0.1) were observed in mixtures of the same composition with *P. a.* at high ratios (75% and 50%) at the medium pollution site. At the later stage of decomposition, the mixtures showed significantly negative nonadditive effects (*P*<0.05) for all the tested ratios at the medium pollution site. For species mixtures of *P. a.* and *N. n.*, significantly positive interactions (*P*<0.05) were found at the mass ratio of 25∶75 for low and medium pollution sites, while weak and negative (*P*<0.1) or insignificant effects were observed for other ratios. No significant differences between the observed and expected mass losses in the species mixtures combining *N. n*. with *P. p.* occurred within all the ratios at the three sites.

**Table 6:** Interaction rates (*r_i_*) of species mixtures at the early stage of decomposition and the later stage of decomposition.

		Early interaction rate			Later interaction rate		
Mixture	Ratio	Low	Medium	High	Low	Medium	High
*P. a.* and *N. n.*	75∶25	−0.026	−0.021	−0.053	−0.027	−0.004°	−0.158
	50∶50	0.015	−0.061	0.025	0.026	−0.001°	−0.026
	25∶75	0.022	−0.017	0.042	0.199*	0.149**	0.073
*P. a.* and *P. p.*	75∶25	−0.075*	−0.096°	−0.048	0.031	−0.067*	0.104
	50∶50	−0.015	−0.038°	−0.090	−0.057	−0.049*	0.185
	25∶75	−0.008	−0.096	−0.013	−0.198	−0.143**	0.047
*N. n.* and *P. p.*	75∶25	0.049	−0.004	0.082	0.026	−0.057	0.141
	50∶50	0.052	0.042	0.099	−0.121	−0.175	0.072
	25∶75	0.020	−0.004	0.069	0.048	−0.125	0.168

° *P*<0.1,

* *P*<0.05,

** *P*<0.01.

Species abbreviations are: *P. a.*, *Phragmites australis*; *N. n.*, *Nelumbo nucifera*; *P. p.*, *Potamogeton pectinatus L*.

### Carbon(C), nitrogen (N) and phosphorus (P) dynamics in decomposing detritus

**Figure 3:**
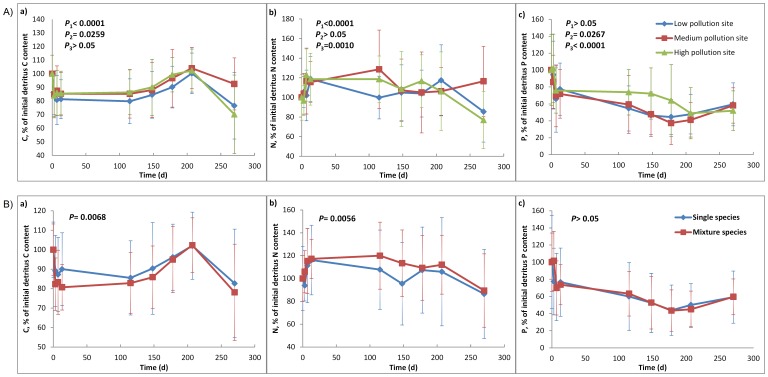
Detritus C, N, and P dynamics. (A) Changes in carbon (a), nitrogen (b), and phosphorus (c) contents (as mean values of the percentage of the initial content for all single and mixed species at each site) during decomposition at the three study sites. (B) Changes in carbon (a), nitrogen (b), and phosphorus (c) contents (as mean values of the percentage of the initial content) between single species samples and species mixtures. Error bars represent ± SD. *P_1_*, *P_2_*, and *P_3_* in (A) indicate significant values during the whole experimental period between low and medium pollution sites, low and high pollution sites, and medium and high pollution sites, respectively. *P* in (B) indicates the significant value between single species samples and species mixtures during the whole experimental period.

As detritus decomposed continuously, detritus N dynamics varied spatially and temporally, and C and P contents in the detritus showed similar trends among the sites (Fig. 3A). Distinct differences in N content were detected between the medium pollution site and the other two sites (*P_1_*<0.0001, *P_3_* = 0.0010). The dynamic patterns of P were different from the patterns of N. P content in the detritus showed a gradual decrease after an early sharp decline period (≤13 days), and the differences in P dynamics among the three sites increased from the third sampling time and declined after the sixth sampling time (Fig. 3A). The C content also exhibited a clear decrease in early stages. Significant differences in C content were found between low and medium pollution sites as well as low and high pollution sites over the experimental period (Fig. 3A, *P*_1_<0.0001, *P_2_* = 0.0259).

The differences in the C, N, and P dynamics between species mixtures and single species suggest that the interactions occurring in species mixtures influence the dynamics of N and C from detritus (Fig. 3B). Although the changes of detritus P in the species mixtures were similar with those in the single species samples (*P*>0.1), significant differences were found for C and N dynamics (*P* = 0.0068, *P* = 0.0056). The N content in the single species decreased sharply after the first few days, while in the species mixtures, it showed a slow increase during the fourth and fifth sampling times. A similar enlarged deviation can be found during the fourth and fifth sampling intervals for C content between single species and species mixtures (Fig. 3B).

## Discussion

### Influence of water chemistry and initial detritus quality on detritus decomposition

In this study, ANOVA analysis showed that site condition is an important factor influencing decomposition. Its effect was further investigated by analyzing relationships between the mass loss rates in response to water nutrients. The difference in water chemistry of the three sites could affect decomposition by influencing microbial compositions and activities, leading to different responses of detritus mass loss among these sites [18], [20] (Table 4). At the low and high pollution sites, changes of pH and inorganic N (NH_4_-N and NO_3_-N) contributed significantly to the variations in detritus mass loss. The results showed that an increasing pH stimulated detritus mass loss at the low pollution site (mean pH = 7.6), while it inhibited detritus mass loss at the high pollution site (mean pH = 8.2). This is consistent with the findings of Suberkropp and Klug [34], who reported that a mean pH of 8.0 was the optimal conditions for enzyme activities on lignin and cellulose decomposition. At the high pollution site, the inhibitory effect of N on decomposition may be caused by NH_4_-N toxicity, which inhibits the growth of some methanogens in a high pH condition [35], [36]. Meanwhile surplus NH_4_-N can impede lignin decay via condensation reactions, forming undegradable structures in plant detritus [37], [38]. One possible explanation for insignificant NO_3_-N effects on detritus decomposition at the high pollution site is the preferential uptake of NH_4_-N instead of NO_3_-N by microbes [39]. It is reported that NH_4_-N tends to inhibit NO_3_-N assimilation when the NH_4_-N concentration is above 0.1 mg L^−1^ [40]. In contrast, significantly negative effect of NO_3_-N on decomposition was observed at the low pollution site. The stronger effects of NO_3_-N than NH_4_-N at this site may be because mean concentration of NH_4_-N (0.089 mg L^−1^) at this site was too low compared with that of the high pollution site and could not affect NO_3_-N assimilation and microbial activities [36], [40]. Elevated NO_3_-N concentration may reduce the rate of detritus decomposition by changing microbial community and reacting with phenolic compounds forming resistant complexes [26], [41], [42]. Additionally, NH_4_-N was highly variable at this site (mean = 0.089, SD = 0.236), which may also contribute to the non-significant effect on decomposition. No correlations between detritus mass loss rates and water N concentrations were found at the medium pollution site, indicating that this site was not under an N-limited or N-exceeded condition [37], [43]. Insignificant relationships between water TP contents and mass loss rates at all of the sites indicate that TP is not an influential indicator of water chemistry in this lake. This was inconsistent with the results of numerous studies in which P has been found to be the limiting nutrient and to significantly influence decomposition rates [14], [22], [27]. However, the high species effects (ANOVA) and the high correlations between detritus P-related indicators and decomposition suggest that, in our study, P content in detritus may be more important to microbe activities and can offset the water TP limitation [24], [28].

The correlation between decomposition and water chemistry provides some support for our first hypothesis. Results of this study showed that detritus decomposition was faster at the high pollution site than the other two sites. However, detritus decomposition did not decrease with mean nutrient levels at the medium and low pollution sites. Temporal changes of water nutrient concentrations at the two sites may influence water chemistry effects on decomposition [44]. For example, although the mean nutrient concentrations were relatively high at the medium pollution site, the initial N availability was lowest at this site. This may cause low activities of microbes and invertebrates, which contribute to decay and fragment, at the early stage of decomposition and slow down the decomposition processes at this site over the whole study period [45], [46]. The lowest mass losses were observed at this site before the frost period and the mean mass losses at the low and the high pollution sites were 16% and 21% higher than the medium pollution site, respectively, at the thirdly sampling time. Additional, larvae and earthworms were found in some litterbags at the low pollution site. The penetration of fauna greatly reduces the size of detritus fragments and accelerates detritus decomposition at this site [47], [48].

Although detritus decomposition observed in our experiments was significantly affected by site chemistry, this effect was weaker than that caused by detritus quality, especially for single species. This supports our second hypothesis that macrophyte species has a stronger effect on detritus decomposition than site conditions. Variations of initial N and P contents in single species (detritus N: SD = 0.399; detritus P: SD = 0.062) in this study are both about 1.7 times of that in mixture samples (detritus N: SD = 0.233; detritus P: SD = 0.027), and are much higher than that in studies which discovered a stronger site effect [14], [23]. Because detritus nutrients were closely related to decomposition (Pearson correlation, Table 5), the high variations of detritus nutrient contents can cause high variations of mass loss, and lead to the relatively strong detritus quality effect. Meanwhile, net P mineralization of detritus and insignificant relationships between TP in the water and detritus decomposition suggest that the microbes involved in decomposition may obtain the majority of their P requirements from the detritus [28].

The best detritus quality indicators explaining species control on detritus decomposition in this study include detritus P content, the C∶N ratio and the C∶P ratio (*r^2^*>0.85, Table 5). Detritus N and P contents were positively related to mass loss, in both the early stage (before frost) and the later stage (after frost), while the initial mass ratios of C∶N, C∶P, and N∶P were good indicators predicting the decomposition process only for the later stage (Table 5). The low correlation between the initial nutrient ratios and decomposition in the early period was mainly due to the fast leaching of water soluble compounds in detritus at this stage [13], [31], [49]. After the fast leaching period, detritus decomposition was mainly controlled by microbial activities which were influenced by detritus quality [31], [49]. This is why good correlations between element ratios and decomposition were observed at the later stage (Table 5). In addition, microbial activities may also lead to the increased net carbon immobilization after the fourth sampling time in this study (Figure 2). Rejmánková and Houdková [14] demonstrated that C∶N ratios in detritus of between 20 and 50 were good predictors that had a strong linear relationship with decomposition rates. Initial C∶N ratios for macrophytes in this study ranged from 20 to 45, which is in agreement with the range suggested above. The initial detritus N∶P ratios reflect nutrient limitation in detritus for decomposition and the shift between N-limited state and P-limited state occurred at detritus N∶P ratios of 30 to 50 [50]. In this study, initial N∶P ratios in detritus were between 10 and 30. Therefore, N limitation could be expected, which means that microbes need to immobilize extra N from the ambient environment.

### Influence of water chemistry and initial detritus quality on species mixtures

Due to the interactions between different plant species in detritus mixture, the observed detritus mass remaining can be deviated from the expected one which is calculated based on the additive decomposition of the composition species. The nonadditive effects in species mixtures suggest that interspecific interaction should be considered to explain decomposition processes in this ecosystem. One mechanism that is responsible for the nonadditive effects is that species with a relatively high nutrient contents can accelerate decomposition of mixtures by providing nutrients to low quality species, and this effect decreases with increases in the proportion of low quality detritus [15], [17], [51]–[53]. The significance of interactions varied with sites and no significant interactions were observed at the high pollution site. This lack of interactions between mixtures is because the nutrient enrichment at the site can lower the C∶N ratio of high detritus quality species and decrease the difference among species, reducing the interactions of mixtures [54].

At the early stage of decomposition, leaching of labile materials from detritus dominate the decomposition process [13], [31], [49], and that was why species mixtures only had weak significant interactions during this period. Specifically, nonadditive effects were only observed in mixtures of *P. a.* and *P. p.* at ratios of 75∶25 and 50∶50 at low and medium pollution sites. Since *P. p.* is a needle detritus which is hard to decompose in the leaching stage [55], low nutrients were supplied from *P. p.* during the early experimental period. Therefore, the stimulatory effects of nutrients provided by high-quality species (*P. p.*) cannot offset the inhibitory effect on microbial activities caused by recalcitrant materials in *P. a.* at the early stage of decomposition [16], [56].

At the later stage of decomposition, nonadditive effects of species mixtures were stronger than those at the early decomposition period (Table 6). The significance and strength of interspecific effects vary with the species composition and evenness, which can cause differences in initial detritus chemistry of macrophytes [33], [51]. For example, in the mixture of *P. a.* and *N. n.* in this study, weak but significant effects (*P*<0.1) were detected when low quality species, *P. a.*, was at a high proportion (75% and 50%) in detritus mixtures. However, with the increase in the proportion of *N. n.*, the interaction rates shifted from negative to positive, and the strengths of the effects were enhanced (*P*<0.01). This result is consistent with the mechanism of nutrient transfer from high detritus quality species to those of low detritus quality [15], [17], [51]–[53]. Additionally, significant differences in the chemistry of *P. a.* and *N. n.* amplified the heterogeneity of the microhabitat in litterbags and increased faunal abundance and biological activities and further accelerated detritus decomposition [30], [51]. Significant, negative interactions (*P*<0.05) were found in mixtures of *P. a.* and *P. p.* for all the ratios at the medium pollution site during decomposition after the frost period. These effects could not be explained solely by nutrients provided by high detritus quality species because a negative interaction was also found in the mixture with a high proportion of *P. p.* (75%). One possible explanation is that the condensation reactions between lignin and N that occurred at the later stage of decomposition impeded lignin decay [56], [57]. Although we did not measure the lignin content in the three macrophytes, previous studies suggest that lignin is higher in *P. a.* and *P. p.* than in *N. n.* [58]–[60]. Therefore, negative interactions were observed in the mixtures of *P. a.* and *P. p.* and of *P. a.* and *N. n.* when *P. a.* was in high proportions (75% and 50%). However, we are not able to explain the negative nonadditive effects existing only at the medium pollution site with relatively high N content. This may be caused by interactions between site and species or differences in microbial compositions, which need to be further studied [16], [30], [56]. No significant interactions (*P*>0.1) were found for mixture samples of *N. n.* and *P. p.*, as these two species had similar initial ratios of C∶N, C∶P, and N∶P [54].

Given that one of our hypotheses is that nonadditive effects vary with the composition and evenness of species at different sites, we have presented evidence that the interactions in mixtures are species and site specific. Although we did not test the correlation between the differences in the detritus chemistry of component species and interaction rates, the increased deviations for the initial nutrient contents of component species can lead to more evident and significant nonadditive effects (Table 5).

### Detritus nitrogen (N), and phosphorus (P) dynamics

Detritus nutrient contents changed greatly during the leaching period. During the first three sampling times, N and P differed in single species and species mixture samples, and varied among the three sites. After the rapid leaching period, the catabolism phase of decomposition becomes more dominant. For detritus N dynamics, strong nonadditive effects usually occurred in the first period of six months, which is consistent with related studies [61]. Net N immobilization was strengthened by the effects of species mixtures compared with the N dynamic in the single species (Figure 2B). This result is in accord with Finzi and Canham [52], who suggest that low quality detritus (low C∶N or low lignin∶N ratios) may impede the net N mineralization until high quality species constitute more than 70% of the mixture. In our study, *P. a.* is characterized with low quality detritus and slow decomposition. Therefore, it may influence N immobilization when it is included in detritus mixtures and change the N content dynamics. Dynamics of detritus P in the mixtures are close to those observed in the single species samples, and no significant acceleration or retention effect is shown in the species mixtures (Figure 2B, *P*>0.05), implying that detritus P may not be an important driver of nonadditive effects in the decomposition of species mixtures in this lake.

Water chemistry has been identified as a regulator for nutrient cycles during the decomposition processes [2], [62]. Previous studies have shown that increased nutrient supply in the ambient environment could stimulate P immobilization [21], [27], which explains the relatively low net P mineralization at the high pollution site during our experiments. However, the values of detritus P content in the three sites became similar to each other at the end of our experiment. It suggests that the net P mineralization was affected little by site differences in the long-term decomposition. On the contrary, deviations of detritus N dynamics among sites occurred at two sampling times (the fourth and the last sampling times). Different microbial compositions and activities at the three sites may be responsible for the above deviations and the significant differences in N dynamics between medium pollution site and the other two sites [25], [46].

In conclusion, we found that both species and site have significant effects on decomposition, and our results indicate a stronger influence of species than site in the investigated eutrophic shallow lake. The initial N and P contents as well as nutrient mass ratios, such as C∶N, C∶P, and N∶P in detritus had significant correlations with detritus mass loss, and the correlations varied with different stages of detritus decomposition. Inorganic N and pH values in the lake water were significant site chemical regulators influencing decomposition processes. Interactions in species mixture samples had considerable impacts on decomposition rates. The strength and significance of these interactions varied among sites and with the species composition. Differences in the chemistry of single species cannot directly explain the nonadditive effects observed in the mixture samples in this study. Species characteristics, together with the nutrient conditions of a site, were probably more responsible for the deviations of the observed decomposition rates from expected rates. Thus, there is a need to further investigate the mechanisms underlying interactions of detritus quality and water chemistry in aquatic plant decomposition.
